# AI‐Guided SERS Defines a Pan‐Cancer Diagnostic Biomarker

**DOI:** 10.1002/advs.202516268

**Published:** 2025-11-16

**Authors:** Cai Zhang, Wen‐Hui Zhao, Duo Zuo, Tianxing Zhou, Wenjing Hou, Lingwei Wang, Shangheng Shi, Yang Yang, Yuanyuan Liu, Shao‐Kai Sun, Li Ren, Zhaoxiang Ye, Dingbin Liu, Dong Li, Xiaoyuan Chen, Jihui Hao

**Affiliations:** ^1^ Department of Radiology Tianjin Medical University Cancer Institute and Hospital National Clinical Research Centre of Cancer Tianjin's Clinical Research Center for Cancer Tianjin Key Laboratory of Digestive Cancer Hexi District Tianjin 300060 China; ^2^ Department of Radiology Tianjin Medical University General Hospital Heping District Tianjin 300052 China; ^3^ Department of Clinical Laboratory Tianjin Medical University Cancer Institute and Hospital Hexi District Tianjin 300060 China; ^4^ Department of Pancreatic Cancer Tianjin Medical University Cancer Institute and Hospital National Clinical Research Center for Cancer Key Laboratory of Cancer Prevention and Therapy Tianjin's Clinical Research Center for Cancer Hexi District Tianjin 300060 China; ^5^ Department of Diagnostic and Therapeutic Ultrasonography Tianjin Medical University Cancer Institute and Hospital National Clinical Research Center for Cancer Hexi District Tianjin 300060 China; ^6^ School of Medical Imaging Division of Medical Technology Tianjin Key Laboratory of Functional Imaging Tianjin Medical University Hexi District Tianjin 300023 China; ^7^ College of Chemistry Research Center for Analytical Sciences State Key Laboratory of Medicinal Chemical Biology Tianjin Key Laboratory of Molecular Recognition and Biosensing Nankai University Nankai District Tianjin 300071 China; ^8^ Department of Diagnostic Radiology Yong Loo Lin School of Medicine National University of Singapore Singapore 119074 Singapore; ^9^ Nanomedicine Translational Research Program NUS Center for Nanomedicine Yong Loo Lin School of Medicine National University of Singapore Singapore 117597 Singapore; ^10^ Clinical Imaging Research Centre Centre for Translational Medicine Yong Loo Lin School of Medicine National University of Singapore Singapore 117599 Singapore; ^11^ Department of Chemical and Biomolecular Engineering and Biomedical Engineering College of Design and Engineering National University of Singapore Singapore 119074 Singapore; ^12^ Institute of Molecular and Cell Biology Agency for Science, Technology, and Research (A*STAR) Singapore 138673 Singapore

**Keywords:** artificial intelligence, exosomes, liquid biopsy, molecular fingerprinting, pan‐cancer biomarker

## Abstract

Early and accurate detection of multiple cancers through a single test remains an unmet clinical need, hindered by current limitations in accuracy, throughput, automation, and multiplexing. Here, we present an AI‐powered SERS chip that combines automated exosome capture with AI‐enabled molecular fingerprinting to accurately distinguish ten common cancer types from a single serum test. The system employs a peptide‐functionalized SERS chip enabling the selective enrichment of exosomes directly from patient serum, enhancing label‐free Raman fingerprint signals. AI‐driven spectral analysis achieved 97.4% accuracy in distinguishing cancer from healthy samples, 97.08% accuracy for early‐stage cancer detection, and 93.89% accuracy in classifying ten common cancer types, including breast, thyroid, esophageal, kidney, pancreatic, duodenal, lung, colorectal, ovarian, and gastric cancers. Crucially, based on molecular profiling, we identified exosomal deoxyadenosine triphosphate as a promising pan‐cancer biomarker consistently upregulated across diverse tumor types. This discovery establishes a potential pan‐cancer diagnostic marker, while the fully automated, scalable platform offers significant promise for clinical translation in early and differential cancer diagnosis.

## Graphical abstract

An AI‐enabled SERS platform integrates automated exosome enrichment with molecular fingerprinting to enable accurate early detection and differential diagnosis of ten common cancers. The system identifies exosomal dATP as a universal Raman biomarker, offering a scalable, noninvasive, and clinically translatable approach for precision oncology.

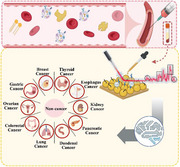

## 1 Introduction

Cancer remains a leading cause of mortality worldwide, and early detection is key to improving survival outcomes.^[^ ^1^ ^]^ Noninvasive liquid biopsy offers significant advantages, including minimal invasiveness, high reproducibility, convenience, and the capacity for real‐time dynamic monitoring, making it a promising approach for early cancer detection and screening. Current liquid biopsy tests primarily rely on broad‐spectrum serum tumor markers such as carcinoembryonic antigen (CEA) and carbohydrate antigen 19‐9 (CA19‐9), as well as cancer‐specific markers.^[^ ^2^, ^3^ ^]^ However, these markers suffer from limited specificity and high false‐positive rates, which restrict their diagnostic accuracy, particularly their inability to differentiate multiple cancer types in a single test.^[^ ^4^ ^]^ Although emerging biomarkers including circulating tumor DNA,^[^ ^5^ ^]^ microRNAs,^[^ ^6^ ^]^ and circulating tumor cells^[^ ^7^ ^]^ show potential for cancer diagnosis, challenges related to sensitivity, procedural complexity, cost, and throughput have thus far limited their clinical translation.

Exosomes, extracellular vesicles ranging from 30 to 150 nm in diameter,^[^ ^8^ ^]^ are abundant in bodily fluids and carry molecular signatures reflective of their cells of origin.^[^ ^9^ ^]^ Tumor‐derived exosomes are enriched with tumor‐associated proteins, nucleic acids, and lipids, and are abundantly present in body fluids, making them promising biomarkers for noninvasive cancer detection via liquid biopsy.^[^ ^10^ ^]^ Recent advances in omics technologies including genomics, proteomics, metabolomics, and lipidomics have enabled detailed molecular characterization of exosomes. Nevertheless, comprehensive profiling often requires the integration of multiple omics platforms, introducing challenges such as technical complexity, high costs, and data integration issues, thereby limiting its feasibility for widespread application.^[^ ^11^, ^12^, ^13^, ^14^, ^15^ ^]^

Raman spectroscopy, renowned for its ability to analyze biological samples without the need for labeling, offers immense potential in biomedical research.^[^ ^16^, ^17^, ^18^, ^19^, ^20^, ^21^, ^22^ ^]^ Especially, Surface‐enhanced Raman Spectroscopy (SERS), which exploits the localized surface plasmon resonance effect of noble metal nanostructures,^[^ ^23^, ^24^, ^25^, ^26^, ^27^, ^28^, ^29^, ^30^, ^31^ ^]^ provides a powerful approach to amplify Raman signals by factors of up to 10^14^,^[^ ^32^ ^]^ enabling the detection of biomolecules at ultra‐low concentrations.^[^ ^33^, ^34^, ^35^, ^36^, ^37^, ^38^, ^39^, ^40^, ^41^, ^42^, ^43^ ^]^ In recent years, the rapid advancement of artificial intelligence (AI) technologies has further enhanced the detection capabilities of SERS, leading to its widespread application in life sciences, including the detection and analysis of exosomes.^[^ ^44^, ^45^, ^46^ ^]^ Conventional SERS‐based approaches generally separate exosome isolation and signal detection into distinct steps.^[^ ^47^, ^48^, ^49^, ^50^, ^51^, ^52^, ^53^ ^]^ Prior to SERS spectra acquisition, the initial isolation of exosomes typically involves labor‐intensive and time‐consuming methods such as ultracentrifugation or immunomagnetic bead capture. This multi‐step process is complex, inefficient, and unsuitable for high‐throughput clinical applications.^[^ ^54^ ^]^ Therefore, the development of integrated SERS platforms enable exosome capture and detection holds profound significance for the early diagnosis and differential classification of multiple cancer types.

**Scheme 1:**
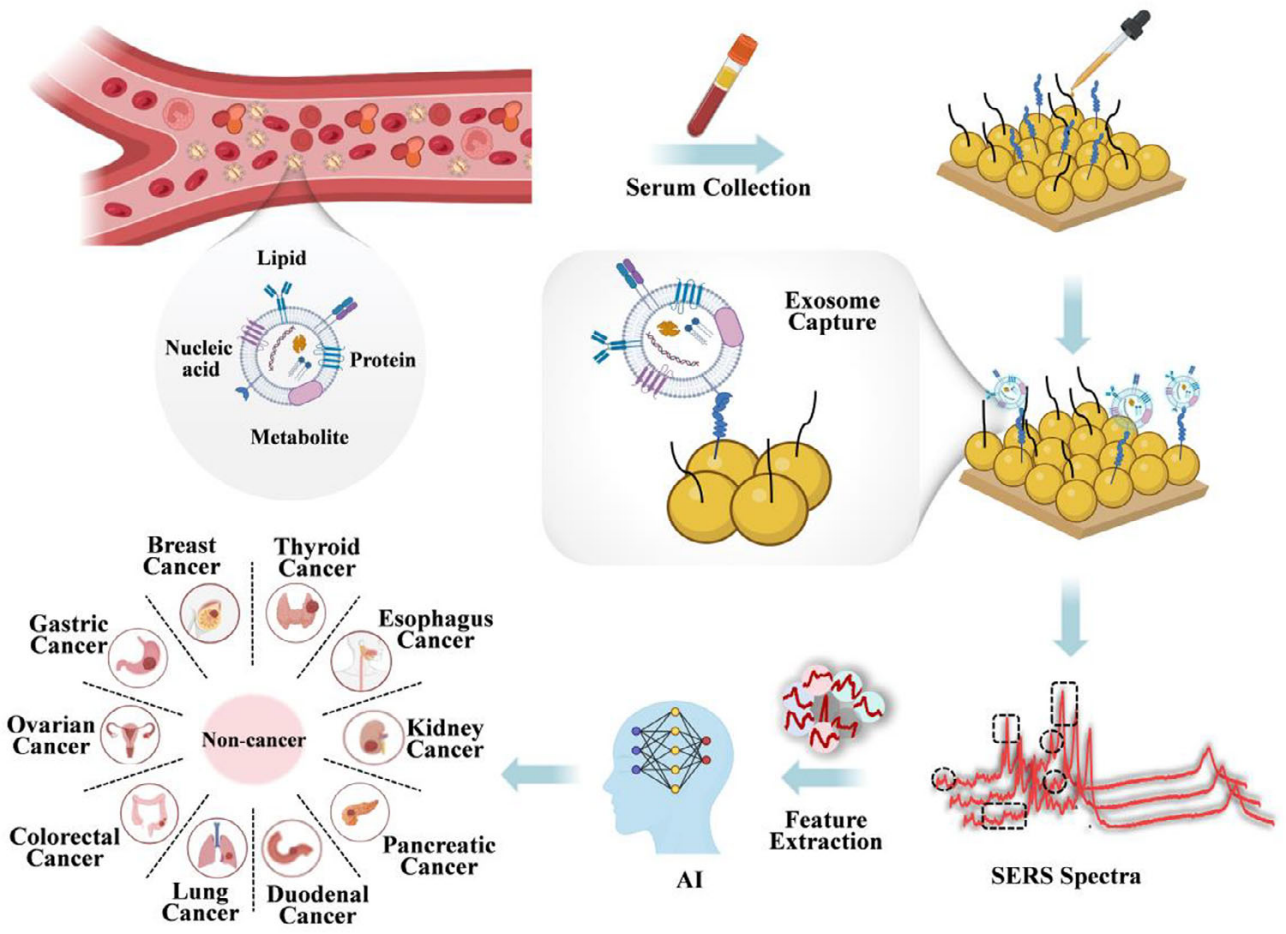
Schematic illustration of an AI‐powered integrated SERS chip for ten‐cancer detection in a single test.

Herein, we show an AI‐powered integrated SERS chip for early detection and differential diagnosis of ten cancer types through exosome molecular profiling. The SERS chip consists of a gold nanoisland substrate functionalized with CP05 peptides, enabling both the selective capture of serum exosomes and label‐free molecular profiling based on SERS (**Scheme** **1**). Combined with AI‐driven spectral analysis, the platform demonstrated 97.4% accuracy in differentiating cancer from healthy samples, 97.08% accuracy in detecting early‐stage cancers, and over 93.89% accuracy in classifying ten common cancer types, including breast, thyroid, esophageal, kidney, pancreatic, duodenal, lung, colorectal, ovarian, and gastric cancer. Crucially, exosome‐based molecular profiling identified a robust Raman fingerprint at 1080 cm^−1^ corresponding to deoxyadenosine triphosphate (dATP), consistently upregulated across multiple cancer types. This molecular fingerprint holds promise as a non‐invasive biomarker for cancer screening across multiple cancer types, providing significant diagnostic value especially for malignancies without established screening markers.

## 2 Results and Discussion

### 2.1 Fabrication and Characterization of SERS Chip

**Figure 1:**
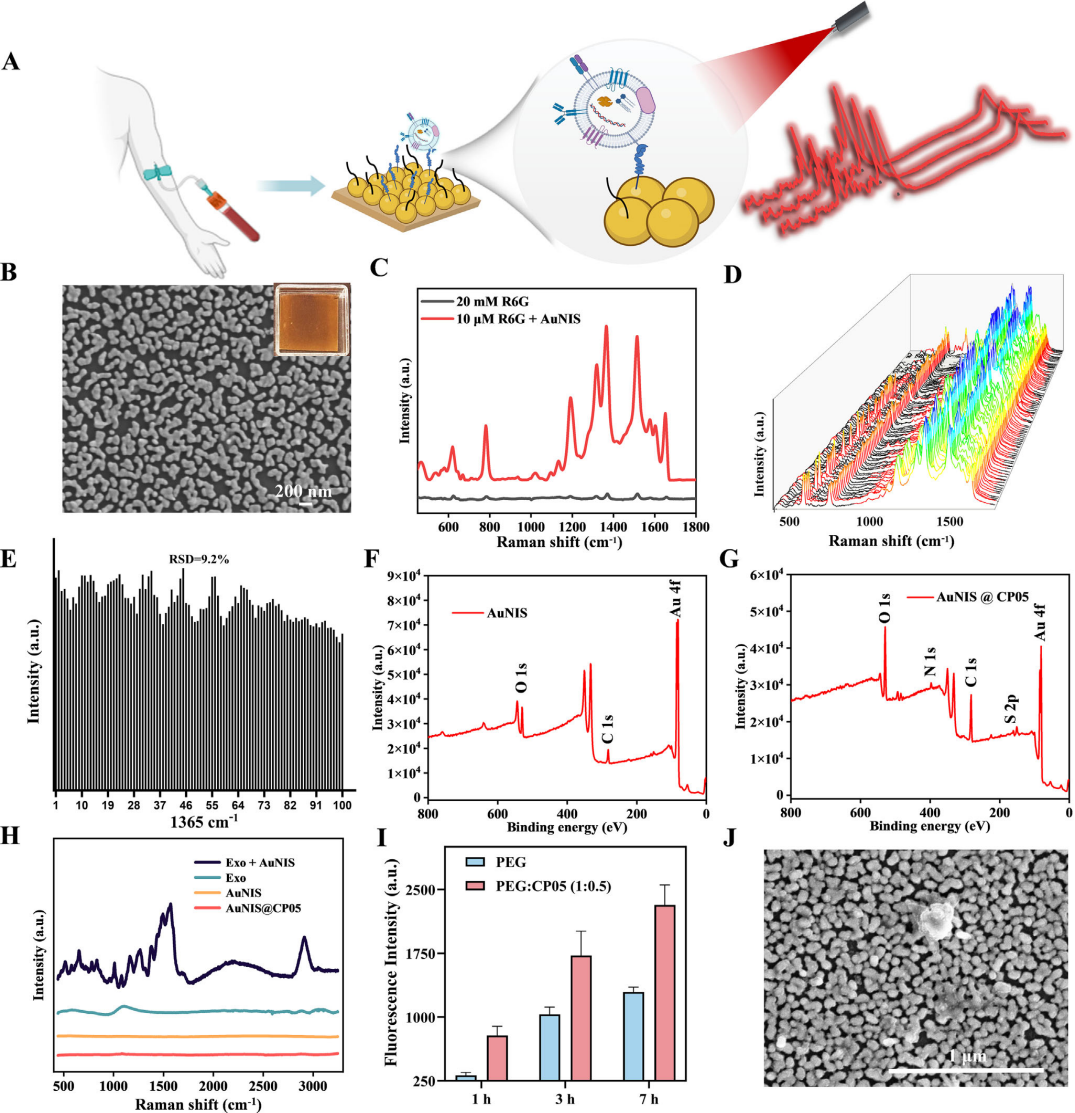
Characterization of the SERS chip. A) Schematic diagram of automated serum exosome capture and SERS analysis. B) SEM image and photo of AuNIS. C–E) Raman enhanced performance and stability of AuNIS. (F‐G) XPS analysis of AuNIS and AuNIS@CP05. H) SERS enhancement performance assessment of AuNIS for exosome detection. I) Exosome capture evaluation of the SERS chip at different time points. J) SEM image of the exosome captured by the SERS chip.

High‐density gold nanoisland substrate (AuNIS) were successfully fabricated on glass substrates via a seed‐mediated growth method for Raman signal enhancement (**Figure** **1**A).^[^ ^55^ ^]^ Scanning electron microscopy (SEM) characterization verified the successful synthesis of AuNIS with irregular geometries, which facilitated the formation of electromagnetic “hot spots” in the nanogap regions (Figure 1B). Rhodamine 6G (R6G) was employed to systematically evaluate the SERS performance of the AuNIS. The enhancement factor (EF) of AuNIS was about 9 × 10^4^ (Figure 1C). The enhancement stability of the substrate was assessed by collecting 100 SERS spectra of R6G, with the signal intensity at 1362 cm^−1^ being evaluated, resulting in an RSD value of approximately 9.2%, indicating superior enhancement stability of the substrate (Figure 1D,E).

CP05 is a functional peptide characterized by a defined amino acid sequence (CRHSQMTVTSRL), which confers high binding specificity toward the exosomal surface marker protein CD63.^[^ ^56^ ^]^ To achieve specific capture of exosomes, the gold nanoisland substrates were co‐incubated with a mixed solution containing CP05 peptide and SH‐PEG. Through the thiol group in its cysteine side chain, the CP05 peptide forms a stable Au‐S bond with the gold surface, leading to a stable modification of the gold substrate. Concurrently, SH‐PEG was also modified on the substrate to form an anti‐fouling layer, effectively suppressing nonspecific adsorption of serum proteins. X‐ray photoelectron spectroscopy (XPS) analysis confirmed the successful immobilization of CP05 (Figure 1F,G), as evidenced by the appearance of S2p binding energy shifts (≈162 eV).^[^ ^57^ ^]^ Different ratios of PEG and CP05 were applied to modify the gold substrate. The BCA assay indirectly confirmed the successful conjugation of the peptide and evaluated the anti‐nonspecific adsorption properties of the functionalized substrates. The results indicated that the CP05:SH‐PEG ratio of 0.5:1 offered the best performance in preventing nonspecific adsorption, and this multifunctional SERS chip was selected for further experiments (Figure S1).

### 2.2 Serum Exosome Capture and SERS Detection

Serum samples used in this study were obtained from 200 non‐cancer controls and 455 pathologically confirmed cancer patients. The expression of exosome surface markers, including CD63, CD81, TSG101 were thoroughly characterized western blotting. These markers are expressed in exosomes derived from both extracellular sources and serum exosomes (Figure S2). In addition, nanoparticle tracking analysis (NTA) was employed to further confirm size distribution of exosomes (Figure S3).

As shown in Figure 1H, the SERS chip exhibited a significant signal enhancement for exosomes, whereas the Raman signal of unenhanced exosome was nearly undetectable. As shown in Figure 1I, the exosome capture performance of substrates functionalized with or without CP05 was assessed, revealing that markedly enhanced binding efficiency was achieved by CP05‐functionalized substrate. After 1 h of exosome incubation, the fluorescence intensity of exosomes captured by the CP05‐modified substrate was found to be approximately 2.5‐fold higher than that of the control. Capture efficiency was observed to increase progressively over time, and at all tested time points, significantly greater exosome enrichment was demonstrated by the CP05 group compared to the unmodified substrate. As shown in Figures 1J and S4, SEM images further confirmed the successful capture of exosomes by the substrate.

Serum samples were subjected to a 10‐fold dilution and processed using the SERS chip for automated exosome enrichment. Unbound residual components were removed by washing with PBS, after which SERS analysis was performed on the captured exosomes. Considering that the Raman signals arise from randomly sampled exosomes within the serum, individual spectra may not comprehensively represent tumor‐associated molecular signatures. To overcome this limitation, a minimum of 200 Raman spectra were collected per sample to enhance the reliability of detecting exosomes originating from cancer.

### 2.3 Cancer Presence Diagnosis

**Figure 2:**
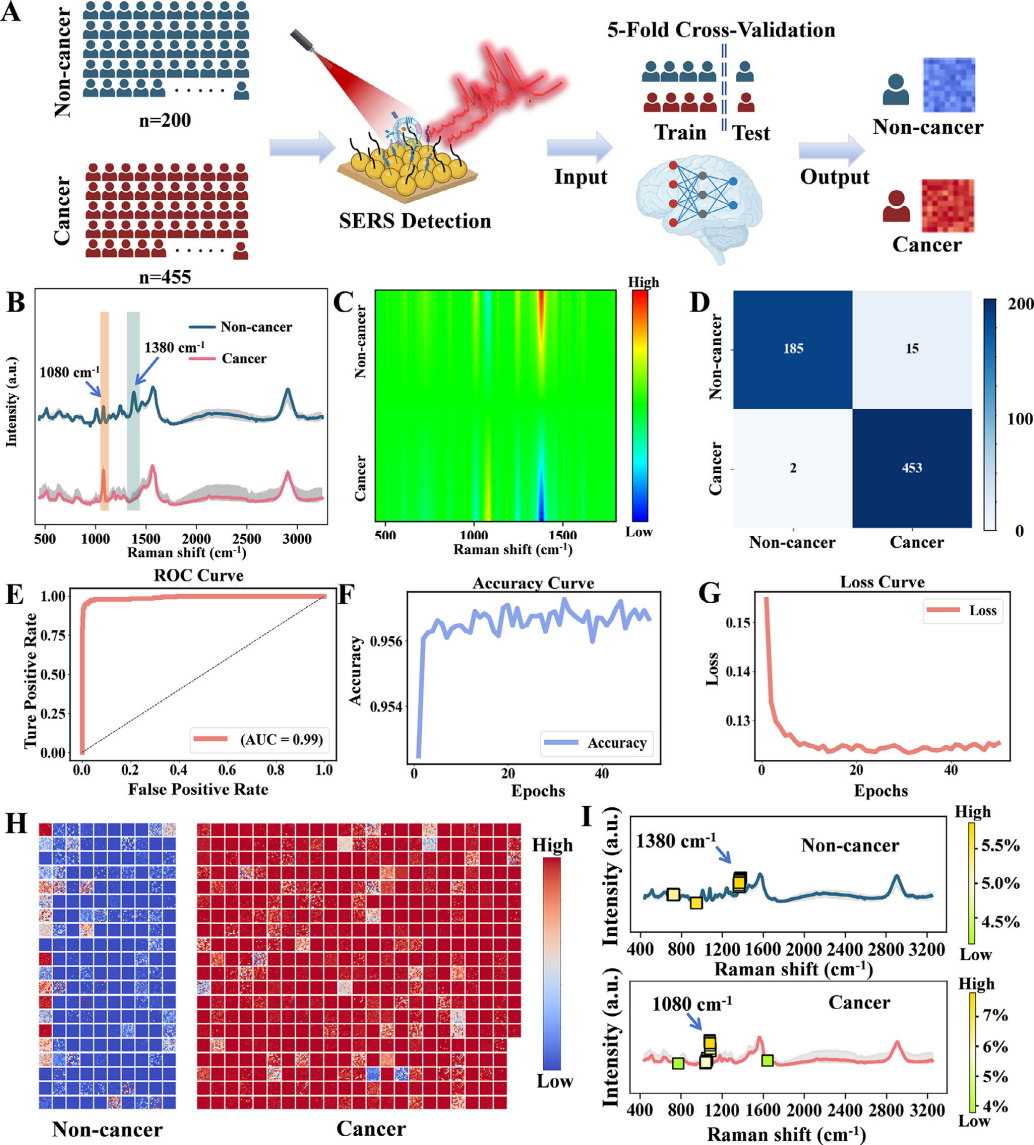
AI‐assisted SERS recognition of cancer presence. A) Diagram showing the process of cancer identification via AI‐SERS. B,C) Comparison of average SERS spectra and heatmaps of serum exosomes from non‐cancer controls and cancer patients (based on 25 spectra per group). D) Confusion matrix of the AI model for classification of non‐cancer individuals and cancer patients. E–G) ROC, Accuracy, and Loss curves for the AI model. H). Heatmap showing the AI model's classification probabilities for SERS spectra derived from non‐cancer and cancer samples. I) Model interpretability analysis of Raman spectral features between cancer and non‐cancer groups. These spectra are the same set of averaged spectra as in Figure 2B, presented separately to illustrate the contribution of individual peaks.

A hybrid AI model combining a convolutional neural network (CNN) and a multilayer perceptron (MLP) was constructed, where the MLP served as a post‐processing module to refine the CNN's output by correcting misclassifications, including false positives and false negatives. To comprehensively evaluate the predictive performance of the CNN model, a five‐fold cross‐validation strategy was implemented. The acquired Raman spectra were analyzed using the artificial intelligence model (**Figure** **2**A). As shown in Figure 2B, the non‐cancer control and cancer samples displayed distinct characteristic SERS spectral profiles. The heatmap of the average spectral differences visually demonstrates the disparities between the two types of spectra (Figure 2C).

The final diagnostic probability for each sample was obtained by averaging the prediction probabilities across all spectra. The confusion matrix illustrated that the model successfully discriminated between cancer patients and non‐cancer controls, with minimal false positives and false negatives, reflecting high classification precision of 97.4% (Figure 2D). The ROC curve exhibited a strong trade‐off between sensitivity and specificity across various decision thresholds, with an AUC value approaching 1, reinforcing the model's outstanding performance (Figure 2E). The accuracy curve showed a progressive improvement in model accuracy during training, stabilizing at an optimal value. The loss curve demonstrated a consistent reduction in the loss function, indicating efficient error minimization and convergence, which confirmed the model's effective learning and stability (Figure 2F,G). In conclusion, the experimental outcomes underscored the model's superior classification ability and robustness in distinguishing cancer patients from non‐cancer controls. The heatmap in the Figure 2H provided a visualization of the classification accuracy for each sample (consisting of 100 spectra), offering further evidence of the AI model's diagnostic performance in cancer detection.

### 2.4 Cancer Molecular Features via AI Model Interpretability

By extracting and analyzing the weight distributions of our deep learning model, we visualized the decision‐making pathways and identified the most discriminative Raman spectral features between cancer and control groups. Feature importance analysis based on model weights revealed that the 1080 and 1380 cm^−1^ bands contributed 7.8% and 5.8% to the model's decisions, respectively, underscoring their pivotal role in distinguishing cancer‐associated molecular phenotypes (Figure 2I). Further spectral analysis indicated a significant downregulation of the 1380 cm^−1^ signal and an upregulation of the 1080 cm^−1^ signal in cancer samples compared to non‐cancer controls. This weight‐based interpretability approach offers valuable insights into the internal mechanisms of the deep learning model and highlights potential molecular biomarkers for cancer detection.

**Figure 3:**
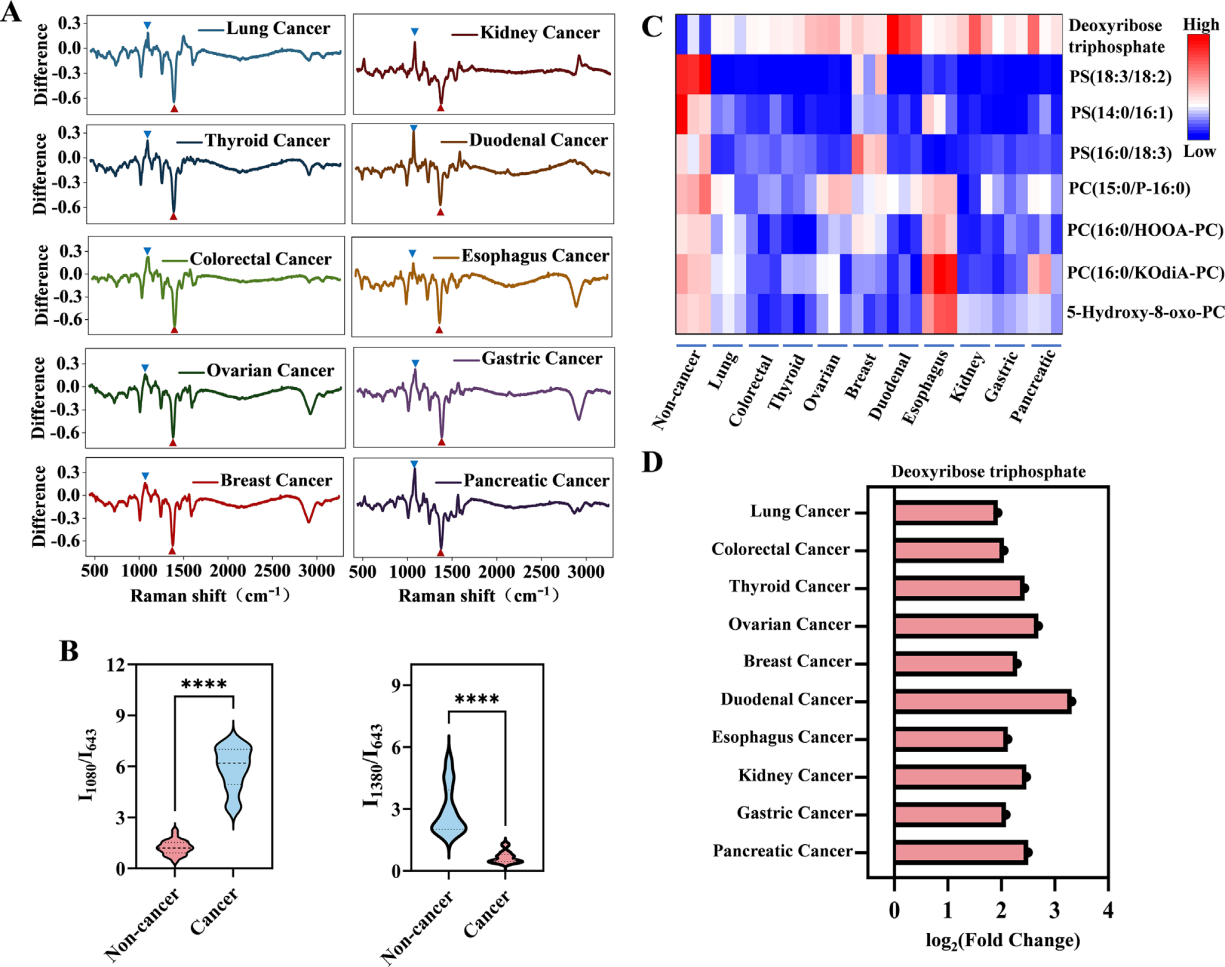
AI model interpretability and comparative analysis between each cancer type and non‐cancer individuals. A) Differential SERS spectra for each cancer type compared to non‐cancer individuals. Blue and red triangles indicate the significantly altered Raman peak at 1080 and 1380 cm^−1^, respectively. B) Comparison of the Raman intensity ratios at 1080 to 643 cm^−1^ and 1380 to 643 cm^−1^ among non‐cancer individuals and cancer samples, based on 25 spectra per group. C) Metabolomics analysis of serum exosomes for each class. D) Comparison of dATP expression in each cancer type relative to the non‐cancer group.

To elucidate specific spectral variations of each cancer type, we performed differential spectral analysis by subtracting the mean Raman spectra of the non‐cancer cohort from those of each cancer group, followed by visualization through differential spectral heatmaps (**Figures** **3**A; S5). This revealed a consistent and marked increase in signal intensity at 1080 cm^−1^ related to dATP and decrease at 1380 cm^−1^ related to phosphatidylserine (PS) across ten tumor types.^[^ ^58^, ^59^ ^]^ The SERS spectra of PS and dATP were also recorded, as shown in Figure S6. These molecular fingerprints exhibited prominent feature importance, substantially influencing model decisions and underscoring their pivotal role as cancer detection markers. The consistent downregulation of PS across diverse tumor types may reflect altered membrane remodeling or dysregulated lipid sorting mechanisms associated with tumor progression.^[^ ^60^, ^61^ ^]^ The observed elevation of dATP in serum exosomes from cancer patients may reflect dysregulation of deoxynucleotide metabolism associated with enhanced cellular proliferation and genomic instability. This imbalance in dNTP pools could potentially promote mutagenesis and contribute to tumor progression.^[^ ^62^ ^]^ This common metabolic alteration observed across multiple cancer types highlights consistent molecular changes in serum exosomes from tumor patients, underscoring their potential as pan‐cancer screening biomarkers with substantial clinical diagnostic significance.

Detailed spectral analysis (Figure 3B) revealed significant differences in Raman intensity ratios of 1080 and 1380 cm^−1^ relative to 643 cm^−1^. The Raman signal at 643 cm^−1^ was chosen as an internal reference due to its relative stability across both cancer and non‐cancer spectra (Figure S7). Metabolomic profiling of serum‐derived exosomes was performed for both cancer patients and non‐cancer controls, revealing significant differences between each cancer type and the control group (Figure S8). A consistent reduction in PS metabolites was observed across multiple cancer types, aligning with the decreased Raman signal at 1380 cm^−1^ in cancer samples (Figure 3C). In contrast, a significant elevation in dATP levels was detected across all cancer types, reflecting enhanced nucleotide metabolism and closely associated with the intensified Raman fingerprint signal at 1080 cm^−1^ (Figure 3C,D). These findings further elucidate the relationship between AI model interpretability and cancer‐associated metabolic features, underscoring the dual role of Raman spectroscopy in oncology research: serving not only as an effective diagnostic classification tool but also as a means to monitor and decode metabolic reprogramming in cancer.

### 2.5 Early Cancer Diagnosis

**Figure 4:**
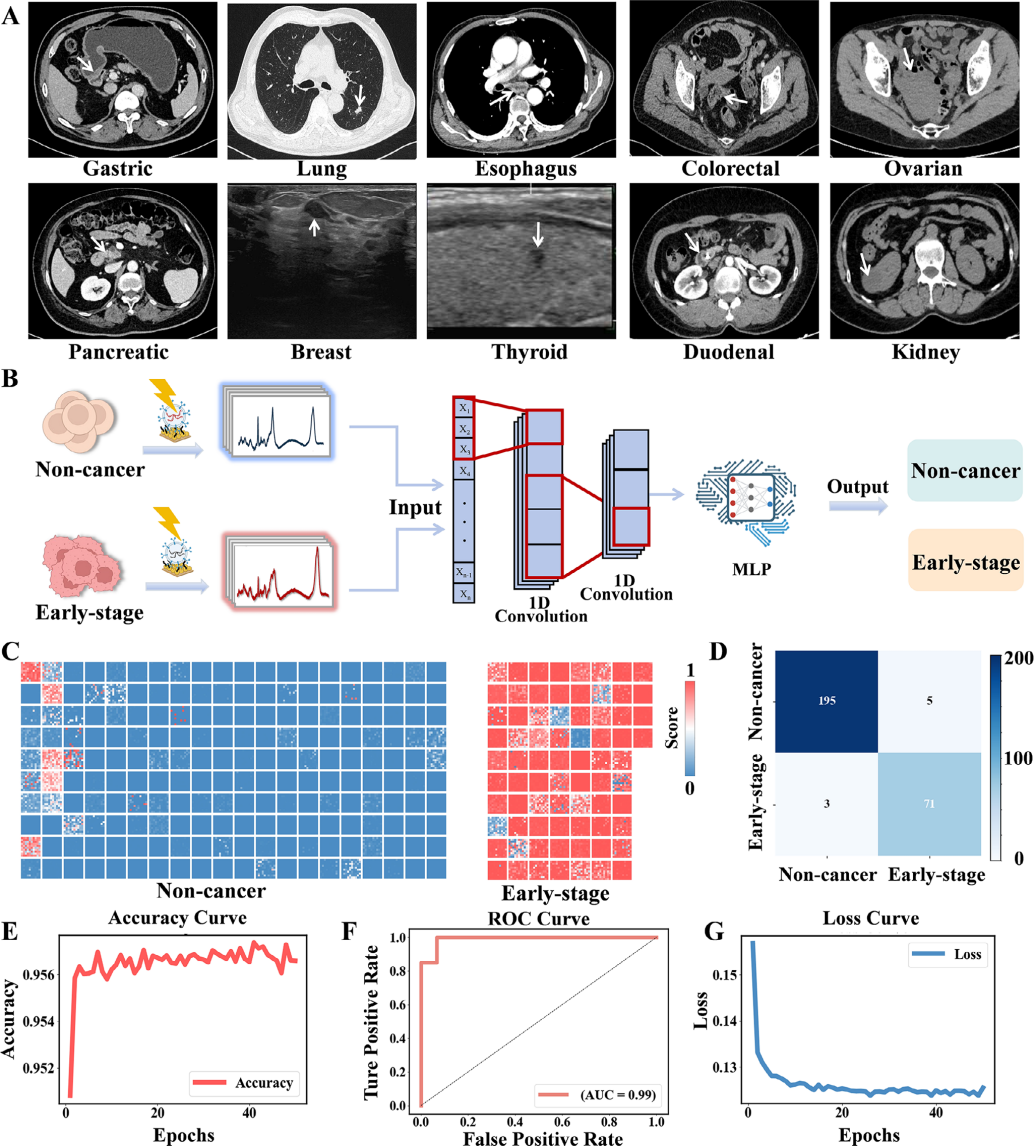
Early cancer diagnosis using AI‐SERS. A) Representative images of ten cancer types, with tumor regions highlighted by white arrows. B) A schematic diagram of an integrated AI model for early cancer diagnosis. C) Heatmap visualization of the AI model's classification probabilities for non‐cancer and early‐stage cancer samples. D) Confusion matrix for classifying non‐cancerous individuals and early‐stage cancer patients. E–G) Accuracy, ROC, and Loss curves demonstrating the classification results of the AI model for identifying non‐cancerous individuals and early‐stage cancer patients.

As shown in **Figure** **4**A, the detection of early‐stage malignancies via imaging is often difficult, as these tumors typically present with subtle or occult characteristics that are not easily detectable. Even when structural abnormalities are observed, differentiating these from benign lesions can be complex, thereby complicating early cancer diagnosis. Additionally, we assessed the classification performance of the AI model in early‐stage cancer patients, with early‐stage cases defined as those with TNM stages T1N0M0 or T2N0M0 (except for ovarian cancer) (Figure 4B; Table S1). For ovarian cancer, early‐stage cases are classified as FIGO stage I, according to the International Federation of Gynecology and Obstetrics (FIGO). The classification probabilities derived from the 100 SERS spectra per sample clearly distinguish between the non‐cancer control group and cancer patients. In the non‐cancer samples, the majority of spectra exhibited a classification probability close to 0, whereas the cancer samples exhibited the inverse trend (Figure 4C). The confusion matrix revealed that the model achieved an accuracy higher than 97.08% in detecting the presence of early‐stage cancer (Figure 4D). The accuracy curve demonstrated stable and high classification performance, reflecting the model's robust predictive reliability. The ROC curve yielded an area under the curve (AUC) of 0.99, underscoring the model's exceptional discriminative ability between cancerous and non‐cancerous samples. Furthermore, the loss curve indicated a continuous reduction in the loss function, signifying efficient convergence and model optimization (Figure 4E–G). These findings validate the AI model's superior diagnostic performance and emphasize its potential as an effective tool for early‐stage cancer detection.

### 2.6 Precise Cancer Type Diagnosis

**Figure 5:**
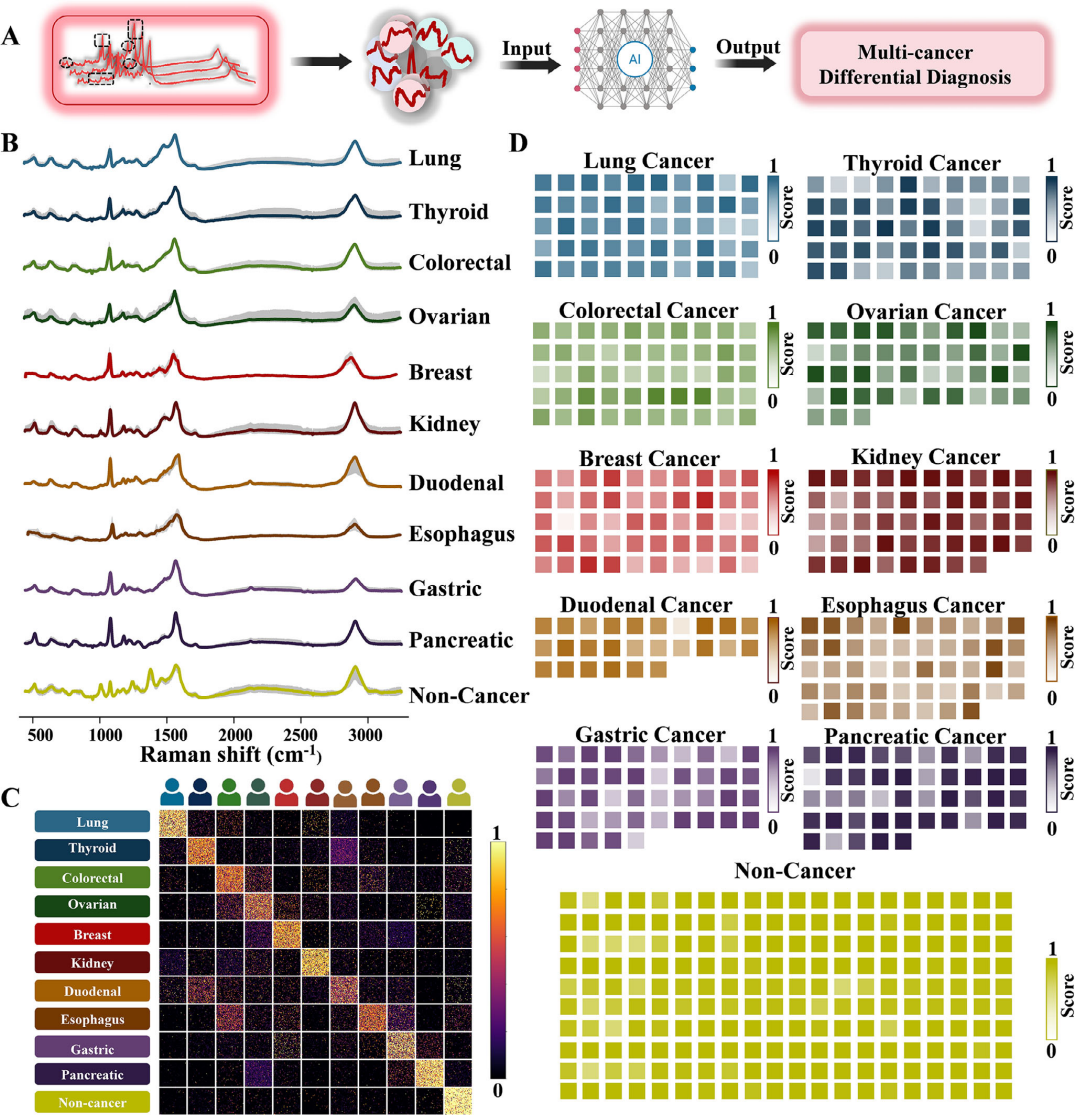
Accurate multi‐cancer diagnosis A) Schematic of multi‐cancer classification. B) Average SERS spectra from different cancer types and non‐cancerous individuals (based on 25 spectra per group). The non‐cancer spectra are the same set of averaged spectra as in Figure 2B, displayed for direct comparison with each cancer spectra. C) Heatmap of the classification probabilities derived from spectra for eleven classes (ten cancer types and non‐cancer group). D) Heatmap visualization of cancer probability for each patient.

Accurate identification of cancer type is as critical as determining cancer presence, forming the basis for precision diagnosis and personalized treatment approaches. Accordingly, we established a multiclass classification system based on a CNN‐MLP ensemble architecture. This system was designed to classify serum samples derived from ten distinct cancer types as well as non‐cancer controls (**Figure** **5**A). Figure 5B presents representative SERS spectra of exosomes isolated from serum samples of each category, highlighting the distinct spectral features that differentiate the groups. Figure 5C presented a visualization of the spectral classification probabilities generated by the model for serum‐derived exosomes from different cancer types and non‐cancer control, with each class represented by the classification probability values of 2 500 individual spectra. The heatmap in Figure 5D presented the visualization of average classification probabilities for each sample, with the majority of instances showing the highest likelihood in their respective class, thereby validating the model's precision and robustness in predictive performance.

**Figure 6:**
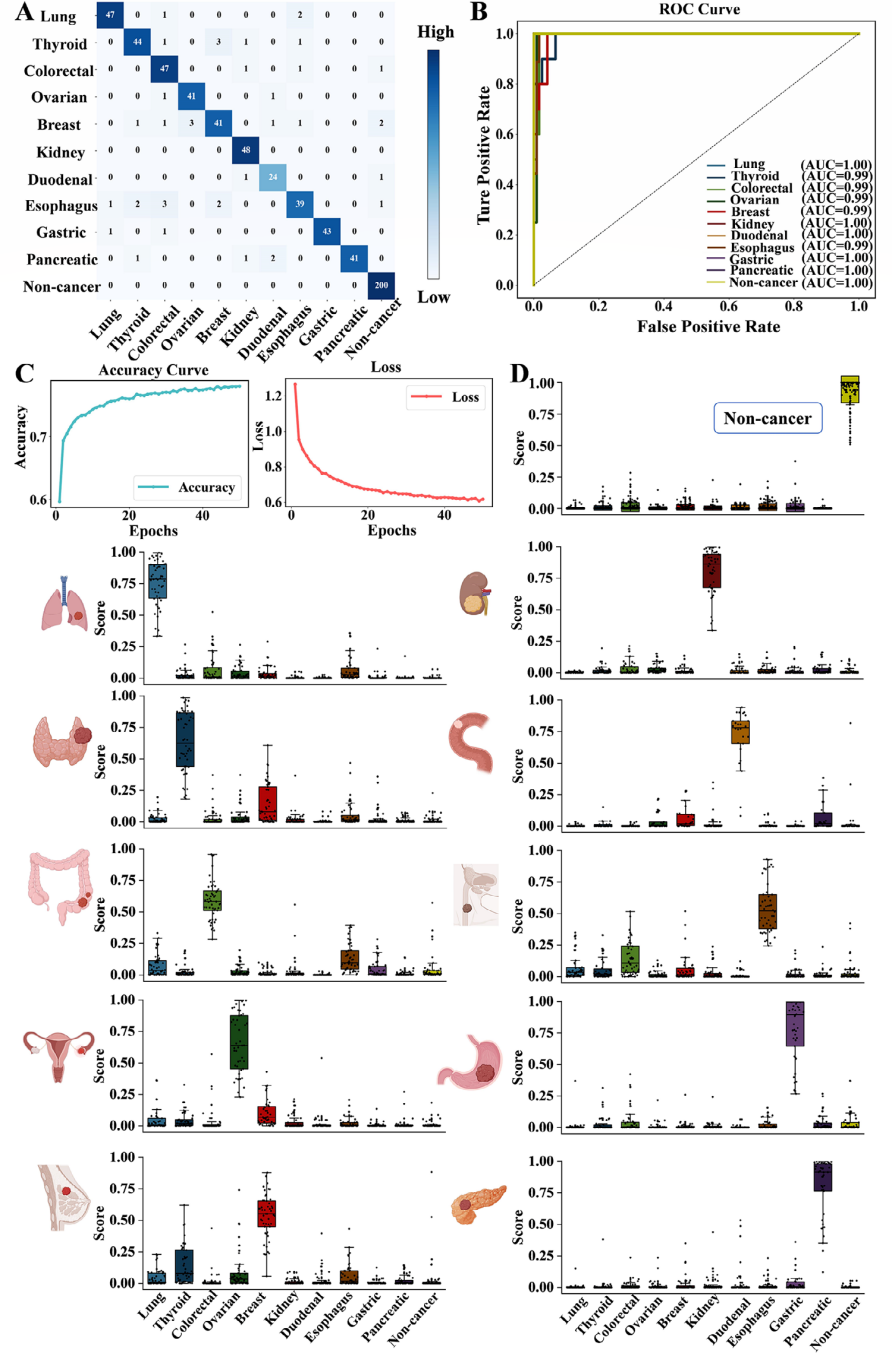
Evaluation of the AI model's precision diagnostic capability. A) Confusion matrix for the 11‐class classification (ten cancer types and non‐cancer individuals). B,C) ROC, Accuracy and Loss curves for the AI model. D) Model's diagnostic probability for each class.

The confusion matrix for the test set illustrated the model's proficiency in accurately categorizing cancer types, with a high proportion of correctly classified instances in each category, yielding an overall classification accuracy of 93.89%, and sensitivity and specificity values of 93.1% and 99.03%, respectively (**Figure** **6**A). Analysis of the ROC curves further confirmed the model's exceptional ability to discriminate among the different cancer types and non‐cancer controls, with AUC values consistently reaching 0.99 or even 1, underscoring its outstanding discriminative power. The accuracy curve demonstrated stable and elevated classification performance, reinforcing the model's robust predictive capacity across various datasets. Additionally, the loss curve exhibited a steady reduction in the loss function throughout the training process, highlighting the model's effective optimization and convergence (Figure 6B,C). Quantitative comparison demonstrated that the mean predicted probability for each sample was substantially elevated within its corresponding ground‐truth class relative to other classes. Specifically, the non‐cancer group exhibited a mean diagnostic probability exceeding that of cancer groups by more than 27‐fold, highlighting the model's robust capacity for multi‐class discrimination and precise classification across diverse categories (Figure 6D). Collectively, these performance metrics substantiate the model's reliability and robustness, confirming its potential as a powerful tool for cancer detection and classification in clinical settings.

## 3 Conclusion

Early cancer detection remains a major challenge in clinical oncology. Exosomes have emerged as promising noninvasive biomarkers due to their early secretion during tumorigenesis and their ability to circumvent limitations of traditional markers such as low abundance and molecular degradation. SERS, while highly sensitive for liquid biopsy applications, is often hindered by the disjointed workflow of conventional approaches, which involve separate exosome isolation and Raman signal acquisition steps. These methods are typically labor‐intensive, time‐consuming, and not amenable to high‐throughput clinical translation. To overcome these limitations, we developed an AI‐powered integrated SERS chip for early detection and differential diagnosis of ten cancer types through exosome molecular profiling. This system employs a CP05 peptide‐functionalized gold substrate for efficient enrichment of serum exosomes and enables rapid acquisition of high‐quality SERS signals using only 200 µL of sample, thereby minimizing sample volume and reagent use. The AI‐driven spectral classifier yielded high diagnostic accuracy for early‐stage cancers (97.08%) and robust differentiation across ten cancer types (93.89%), with sensitivity and specificity exceeding 93.1% and 99.03%, respectively.

Interpretability analysis reveals that the Raman intensity at 1080 cm^−1^, attributed to dATP, serves as a key molecular fingerprint consistently elevated across all tumor types examined. Metabolomic analysis revealed elevated dATP levels in serum exosomes from cancer patients, providing indirect evidence for these findings and reflecting dysregulated deoxynucleotide metabolism associated with increased cellular proliferation and genomic instability. Collectively, these results underscore the potential of this integrated platform as a simple, efficient, and clinically translatable noninvasive approach for cancer detection.

## 4 Experimental Section

### Chemicals and Materials

Chloroauric acid trihydrate (HAuCl_4_·3H_2_O) and hydroxylamine hydrochloride (NH_2_OH·HCl) were purchased from Tianjin Heowns Biochemical Technology Co., Ltd. (China). The CP05 peptide was obtained from GL Biochem Ltd. (Shanghai, China). Ammonia solution (30%), sodium borohydride (NaBH_4_), and thiol‐terminated polyethylene glycol (SH‐PEG, molecular weight 550) were acquired from Aladdin Biochemical Technology Co., Ltd. (Shanghai, China).

### Fabrication of Gold Substrates

Glass slides were first sequentially ultrasonicated in acetone, isopropanol, and methanol for 5 min each, followed by thorough rinsing with ultrapure water. The cleaned glass slides were then immersed in 10 mL of aqueous HAuCl_4_ solution (3 mm), to which an appropriate volume of 30% ammonia solution was added. The mixture was vigorously shaken for 2 min to facilitate complex formation. Following this, the substrates were rinsed three times with ultrapure water and transferred into 15 mL of a freshly prepared NaBH_4_ (1 mm) solution to reduce the gold precursor and generate gold seeds. After reduction, the slides were rinsed again and immersed in a growth solution consisting of equal volumes of HAuCl_4_ (1.5 mm) and hydroxylamine hydrochloride (1.5 mm). The solution was gently agitated until the gold nanoparticle layer was formed on the substrate surface.

### Characterization

SEM images were acquired using a Gemini 300 system (Zeiss, Germany). X‐ray photoelectron spectroscopy (XPS) was performed with the Axis Ultra DLD system (Kratos Analytical Ltd., UK). High‐resolution scanning electron microscopy (HRSEM) images were obtained using an Apreo S LoVac system (FEI, Czech Republic). Exosomes were characterized using nanoparticle tracking analysis, Western blotting, and transmission electron microscopy. NTA was conducted using a Nanosight NS300 system (Malvern Panalytical, UK).

### Gold Substrate Functionalization

The CP05 peptide was immobilized on the gold substrate via Au‐S bonding. Furthermore, to optimize the anti‐nonspecific adsorption properties of gold substrates while enabling exosome‐specific capture, we functionalized the gold surfaces by co‐incubation with varying molar ratios of CP05 peptide to SH‐PEG (0:1, 0.5:1, 1:1, and 2:1) at 25°C for 4 h with gentle shaking (40 rpm). Following modification, the substrates were thoroughly rinsed three times with ultrapure water to remove unbound molecules. The surface peptide conjugation efficiency was quantitatively assessed using the BCA protein assay. To evaluate nonspecific adsorption resistance, modified substrates were incubated with 5% BSA solution under identical conditions (25°C, 40 rpm, 1 h), washed extensively, and the adsorbed BSA was quantified using the BCA method, thereby systematically characterizing both the modification efficiency and anti‐fouling performance of each functionalization condition. The gold substrate without any functionalization was used as the control.

### Isolation of Exosomes

Cell culture supernatants were initially centrifuged at 2000 g for 20 min to remove residual cells, followed by centrifugation at 10 000 g for 30 min to eliminate cellular debris. The resulting supernatant was filtered through a 0.22 µm membrane to exclude large extracellular vesicles. The filtrate was then subjected to ultracentrifugation at 1 00 000 g for 70 min to pellet exosomes. After discarding the supernatant, the exosome pellet was resuspended in PBS and further purified by a second round of ultracentrifugation under the same conditions. Finally, the supernatant was removed, and the purified exosome pellet was gently resuspended in 100 µL of PBS.

### Exosome Capture

To systematically evaluate the specific capture efficiency of the functionalized gold substrates, exosome samples were first fluorescently labeled with the membrane dye DiO. Following labeling, the samples were purified by centrifugation using 100 kDa molecular weight cutoff ultrafiltration tubes to remove unbound dye. The purified exosomes were then resuspended in phosphate‐buffered saline, and the initial fluorescence intensity (F_0_) was measured. Gold substrates functionalized with CP05 and SH‐PEG at a molar ratio of 0.5:1 were used as the experimental group, while substrates modified with SH‐PEG alone served as the negative control. Each substrate was placed in a separate culture dish and incubated with 2 mL of DiO‐labeled exosome suspension under standard culture conditions. Supernatants were collected at 1, 3, and 7 h, and the fluorescence intensity at each time point (F_t_) was determined using a fluorescence spectrophotometer. The capture effect was assessed by calculating F_0_‐F_t_ at each time point.

### Serum Exosome Capture and SERS Detection

A total of 655 serum samples were obtained, including 200 from non‐cancer controls and 455 from individuals diagnosed with cancer. First, patient serum was centrifuged at 10 000 g for 30 min to remove cellular debris. The supernatant was collected, diluted 1:10 with phosphate‐buffered saline, and filtered through a 0.22 µm membrane to remove large extracellular vesicles. The resulting solution was applied to SERS chips. The samples were incubated at 25°C under gentle agitation (40 rpm) for 7 h, allowing efficient and sufficient capture of serum‐derived exosomes by the functionalized SERS chip. Following incubation, the SERS chips with captured exosomes were gently rinsed with PBS to remove unbound components. Raman spectra were acquired using a Zolix‐RTS2 Raman spectrometer equipped with a 100× objective lens. The excitation laser power was set to 1.3 mW. An integration time of 2 s per scan was employed for all measurements. The spectrometer was calibrated using the characteristic silicon peak to ensure the accuracy and consistency of spectral measurements. For each sample, at least 200 spectra were randomly collected.

### Deep Learning Algorithm

To prepare the Raman spectra for artificial intelligence‐driven analysis, spectra data underwent a standardized preprocessing pipeline consisting of baseline correction, cosmic ray removal, Gaussian smoothing, and Min‐Max normalization (scaling to the 0‐1 range). This process was critical for enhancing spectral clarity and ensuring the extraction of meaningful biochemical information embedded in intrinsic spectral patterns.

A deep learning model was designed to address multi‐class classification tasks. Initially, a convolutional neural network was employed for the extraction of local features from the input data. The model begins with a one‐dimensional convolutional layer (64 filters, kernel size 3, stride 1) to capture relevant features. To mitigate overfitting and enhance generalization, a Dropout layer was applied. This is followed by a max‐pooling layer (pool size 2, stride 2) that downscales the feature maps, reducing both the dimensionality and computational complexity. The processed features are then flattened into a one‐dimensional vector, which is passed through fully connected layers for further abstraction. To improve classification performance, the output class probabilities from the CNN are fed into a multilayer perceptron (MLP) for further refinement. The MLP consists of two hidden layers, each with 64 neurons, and employs the ReLU activation function. The final output is generated through the Softmax activation function, producing the predicted probabilities for each class. This step significantly enhances the model's performance on multi‐class classification tasks by refining the feature representations.

To evaluate the model's generalization capability, five‐fold cross‐validation was implemented. In each fold, the dataset was split into training and validation subsets. The model's parameters were optimized based on loss and accuracy metrics computed on both subsets, ensuring reliable and robust evaluation. Performance metrics such as confusion matrix, accuracy, and ROC curves were generated for each fold to assess the model's ability to generalize across different data splits. This process effectively combats overfitting and improves the model's robustness in handling diverse data distributions. The model was trained for 50 epochs with a batch size of 64. To mitigate overfitting, a learning rate scheduler with a patience parameter set to 5 was employed, allowing for dynamic adjustment of the learning rate during training, thus improving efficiency. After each fold, model performance was evaluated using confusion matrices, accuracy, and ROC curves, demonstrating its effectiveness for both spectral‐level and patient‐level classification tasks. All artificial intelligence workflows, including data preprocessing, model training, and evaluation, were conducted in Jupyter Notebook (version 7.1.4) using Python (version 3.8.5). The models were implemented using PyTorch (version 2.2.2+cu121,) and scikit‐learn (version 1.4.23.10.9). All packages were installed via pip from the Python Package Index (PyPI).

### Statistical Analysis

All experimental data are presented as the mean ± standard deviation (SD). Statistical analysis was performed using the t‐test, with a significance threshold set at *p* < 0.05 (^*^), *p* < 0.01 (^**^), *p* < 0.001 (^***^), and *p* < 0.0001 (^****^). To further validate the results obtained from the SERS analysis, non‐targeted metabolomics was employed as a complementary approach.

## Conflict of Interest

The authors declare no conflict of interest.

## Supporting information



Supporting Information

## Data Availability

The data that support the findings of this study are available in the supplementary material of this article. Bio‐Render.com was used in Scheme 1, Figure 1A, Figure 2A, Figure 4B, Figure 5A, Figure 6D and TOC.
