# Supplementary material for: AI‐Guided SERS Defines a Pan‐Cancer Diagnostic Biomarker
Source: Adv Sci (Weinh). 2025 Nov 16;13(7):e16268. doi: 10.1002/advs.202516268 (PMC12866722; doi:10.1002/advs.202516268)
Supplement: Supplementary file 1 — Supporting Information [file ADVS-13-e16268-s001.docx]

Supporting Information

**AI-Guided SERS Defines a Pan-Cancer Diagnostic Biomarker**

*Cai Zhang^†^, Wen-Hui Zhao^†^, Duo Zuo^†^, Tianxing Zhou, Wenjing Hou, Lingwei Wang, Shangheng Shi, Yang Yang, Yuanyuan Liu, Shao-Kai Sun, Li Ren, Zhaoxiang Ye, Dingbin Liu, Dong Li^*^, Xiaoyuan Chen^*^ and Jihui Hao^*^*

*Correspondence should be addressed to dr_lidong@tmu.edu.cn (Dong Li), chen.shawn@nus.edu.sg (Xiaoyuan Chen), and haojihui@tjmuch.com (Jihui Hao).

Supporting Information consists of Figure S1 to S8 and Table S1


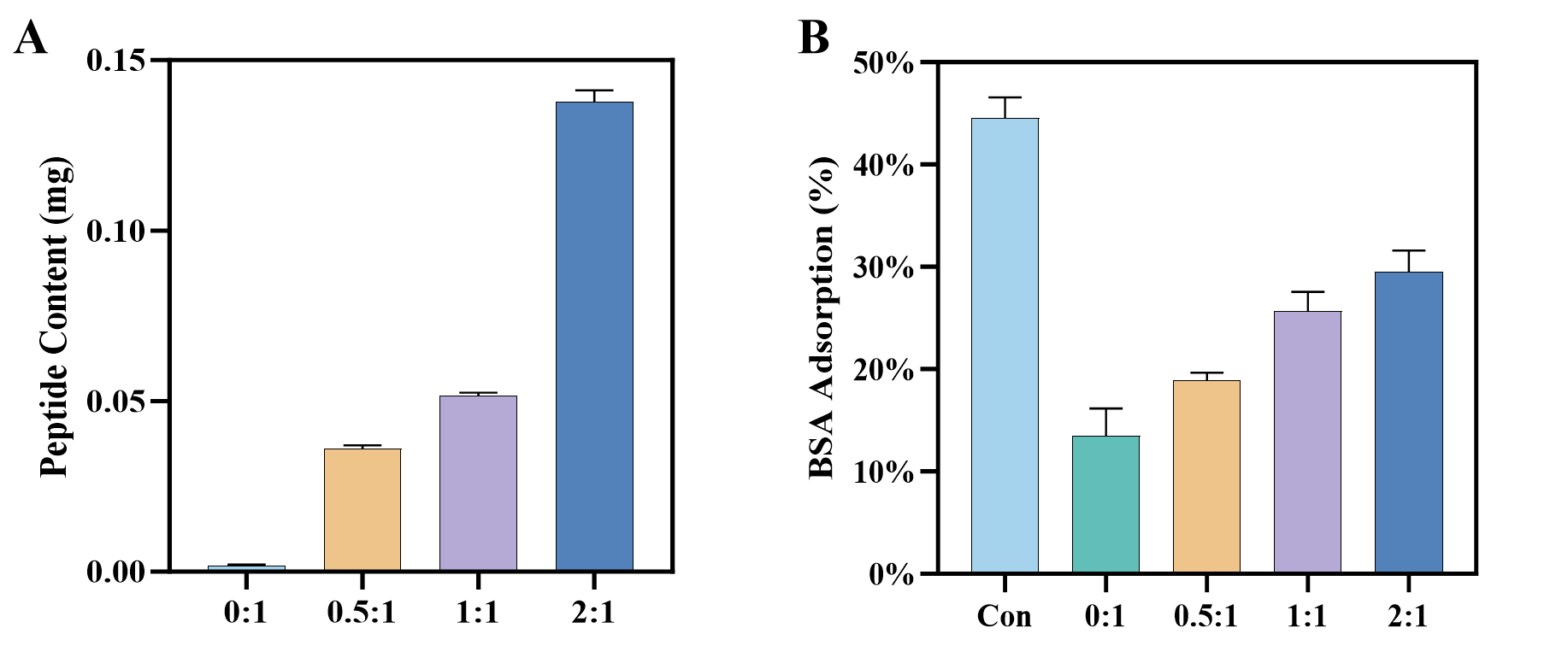


**Figure S1.** Assessment of SERS chip functionalization and minimizing nonspecific protein binding. (A) Evaluation of CP05 peptide immobilization on gold substrates with varying CP05/PEG functionalization ratios. (B) Assessment of nonspecific BSA adsorption on substrates with varying PEG/CP05 ratios; unmodified substrates served as the control.


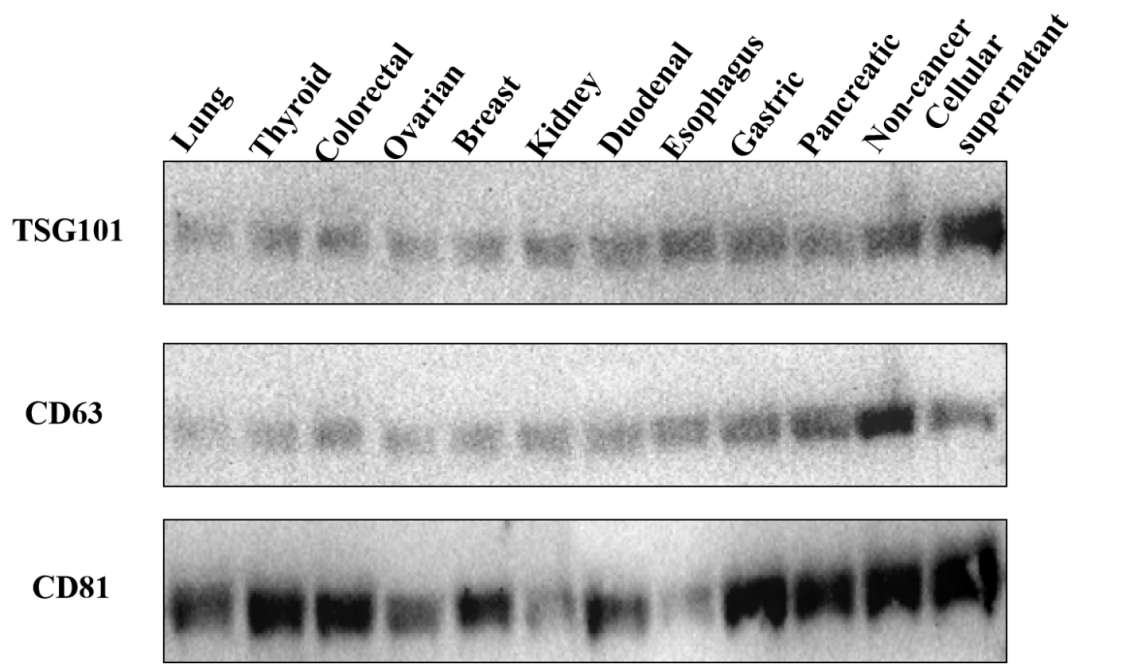


**Figure S2.** Western blotting analysis of the expression of CD63, CD81, and TSG101 on the surfaces of exosomes derived from serum and cells.


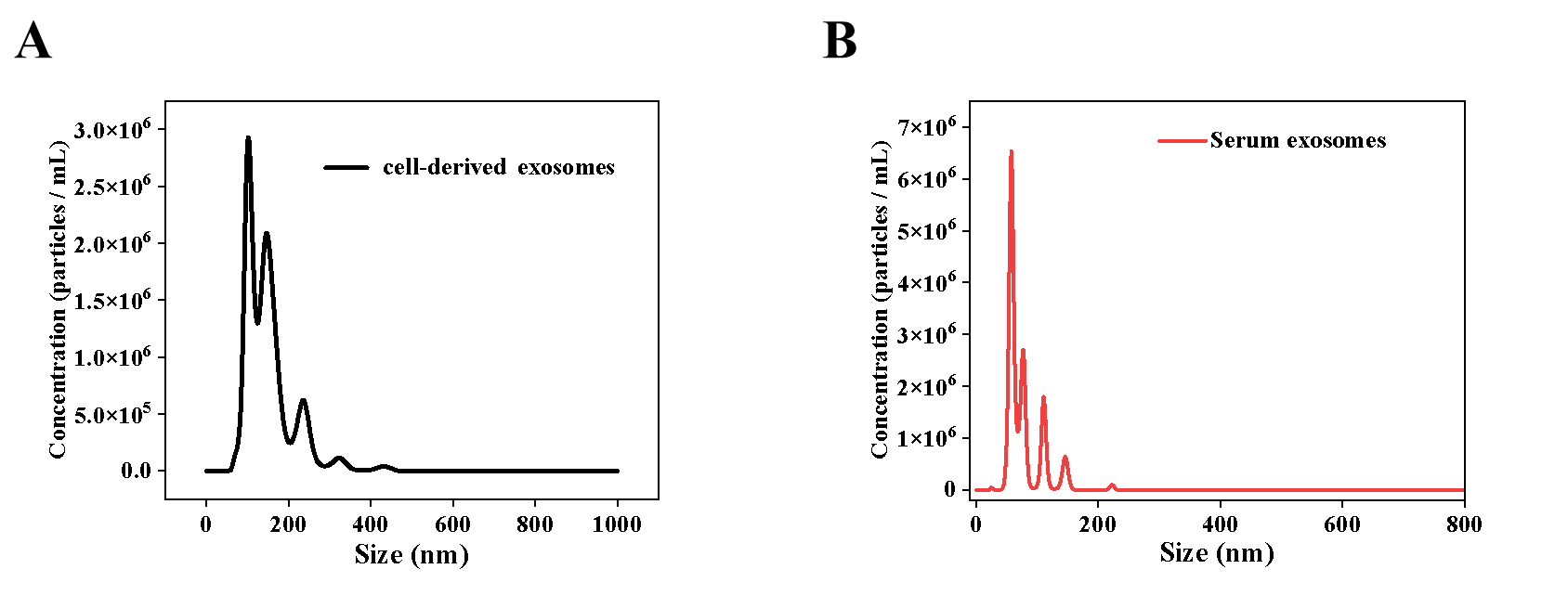


**Figure S3.** Nanoparticle tracking analysis (NTA) of exosomes isolated from cell (A) and Serum (B).


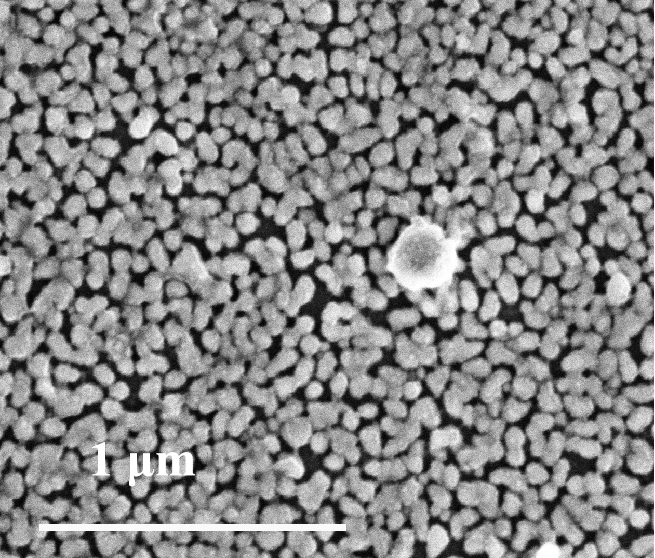


**Figure S4.** SEM image of cell-derived exosome captured by the SERS chip.


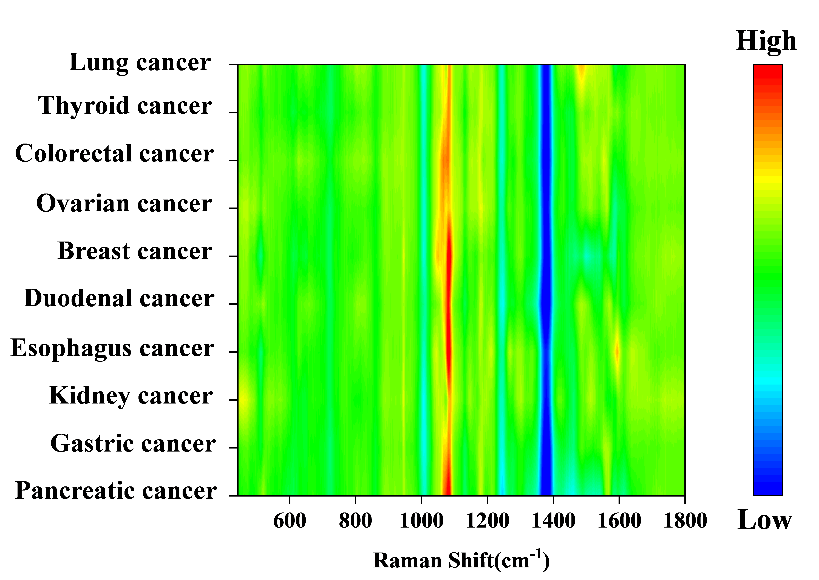


**Figure S5.** Spectral difference heatmap between each cancer type to the non-cancer group.


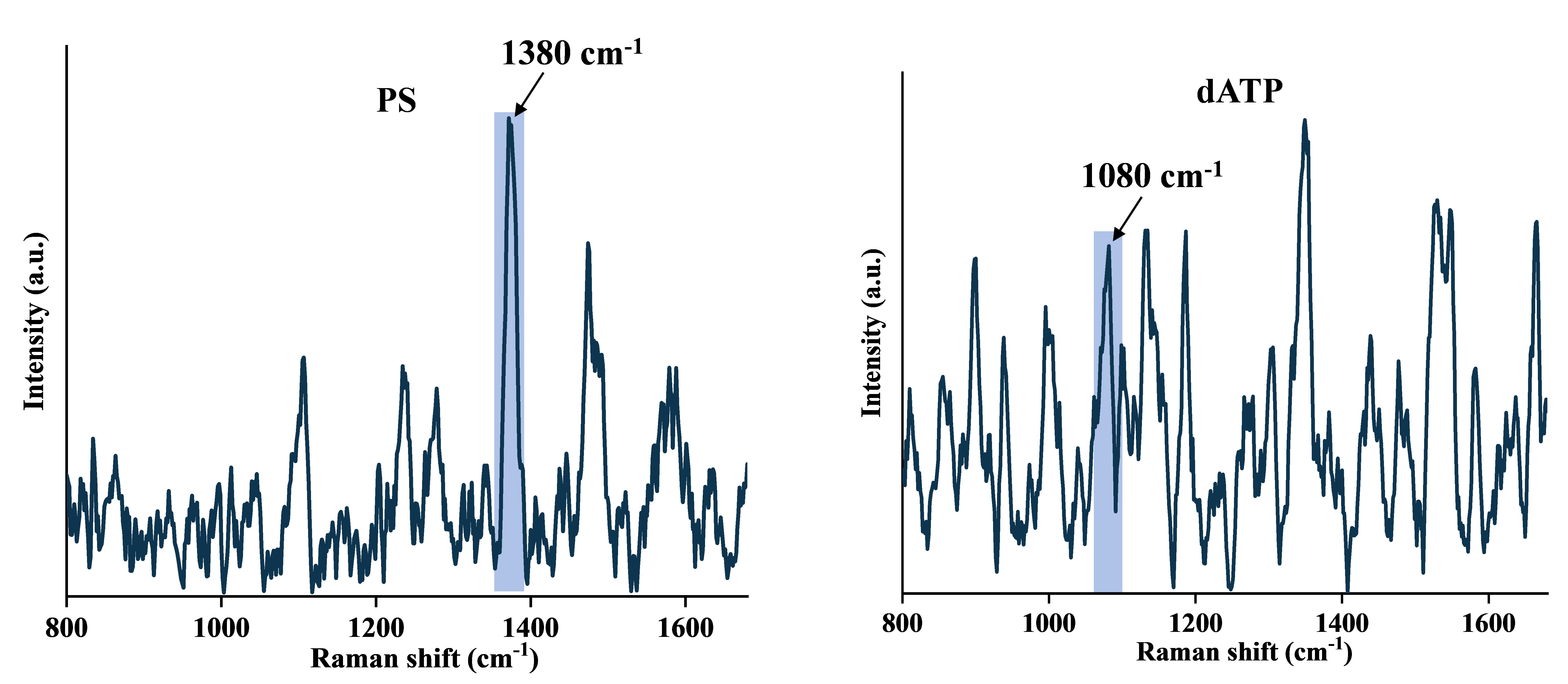


**Figure S6.** Surface-enhanced Raman spectra of phosphatidylserine (A) and deoxyadenosine triphosphate (B).


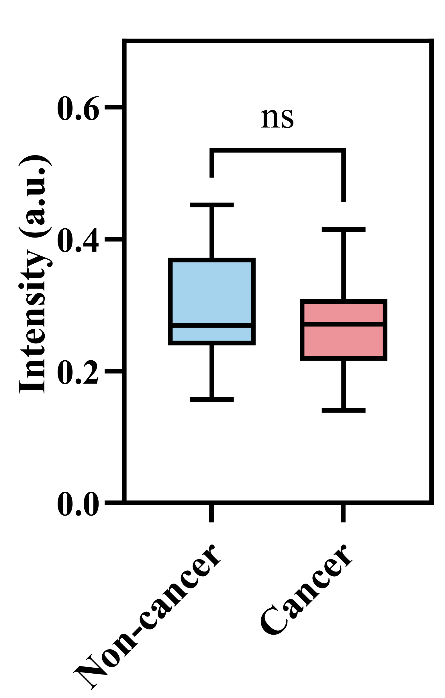


**Figure S7.** Comparison of Raman signal intensity at 643 cm^-1^ between cancer and non-cancer groups.


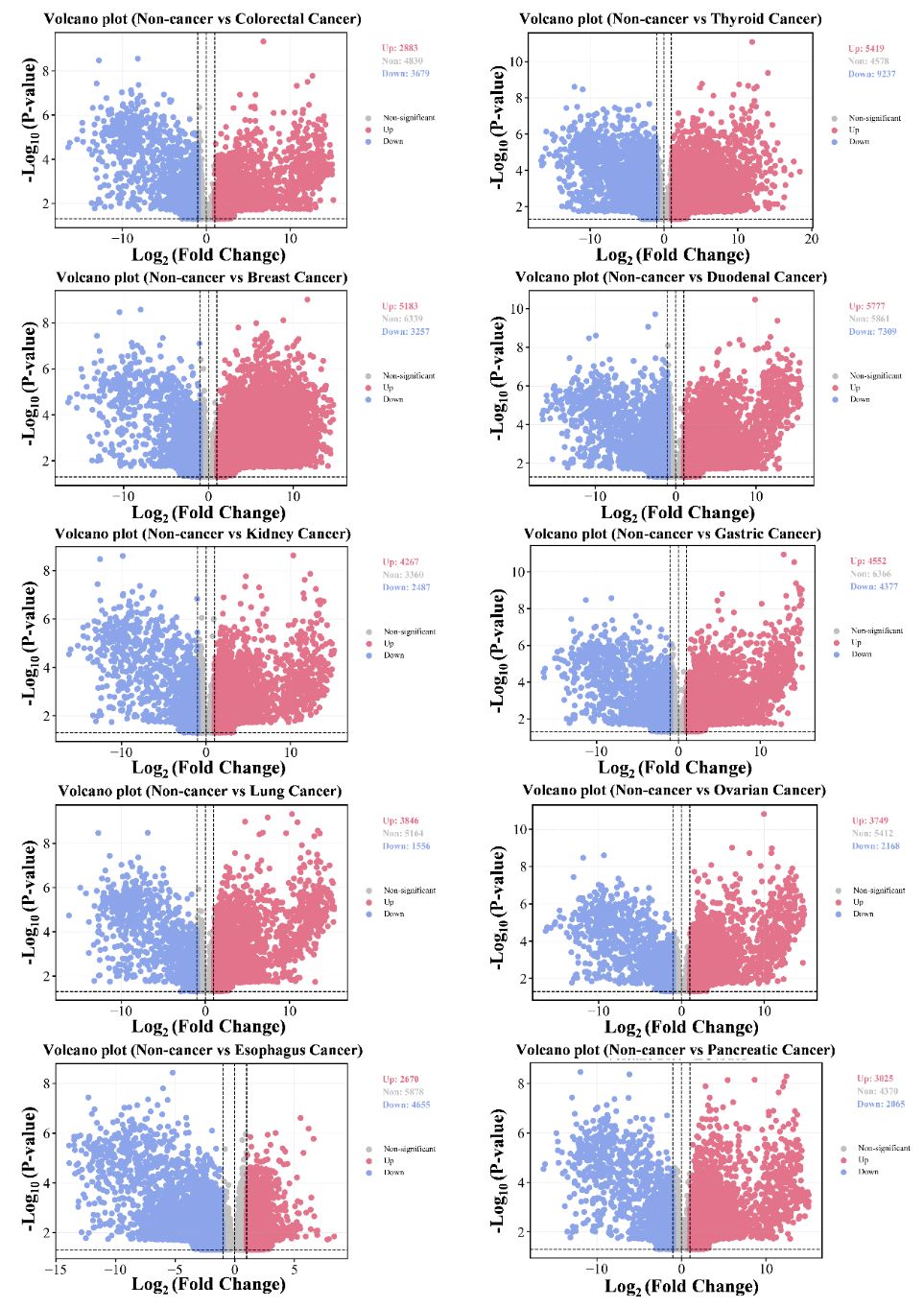


**Figure S8.** Volcano plots of metabolomic profiles for each cancer type compared to the non-cancer group. The red dots indicate significantly upregulated metabolites (log_2_ (fold change) ≥ 1; -log_10_ (P-value) ≥ 1.3), the blue dots indicate significantly downregulated metabolites (log_2_ (fold change) ≤ -1; -log_10_ (P-value) ≥ 1.3), and gray dots represent metabolites with no significant change (-1 < log_2_ (fold change) < 1). The metabolites exhibiting statistical significance (P < 0.05; -log_10_ (P-value) ≥ 1.3) are visualized in the volcano plots.

**Table S1.** Early-stage patients from 10 cancer types.

|  | Lung Cancer | Thyroid Cancer | Breast Cancer | Esophagus Cancer | Kidney Cancer | Duodenal Cancer | Gastric Cancer | Colorectal Cancer | Pancreatic Cancer | Ovarian Cancer |
| --- | --- | --- | --- | --- | --- | --- | --- | --- | --- | --- |
| T_1_N_0_M_0_ | 4 | 16 | 3 | 2 | 20 | 2 | 7 | 0 | 0 | (FIGO I) 8 |
| T_2_N_0_M_0_ | 0 | 0 | 1 | 1 | 2 | 2 | 3 | 1 | 2 | **\** |
